# Assessment of the Morphological, Biochemical, and Kinetic Properties for *Candida rugosa* Lipase Immobilized on Hydrous Niobium Oxide to Be Used in the Biodiesel Synthesis

**DOI:** 10.4061/2011/216435

**Published:** 2011-08-16

**Authors:** Michele Miranda, Daniele Urioste, Livia T. Andrade Souza, Adriano A. Mendes, Heizir F. de Castro

**Affiliations:** ^1^Engineering School of Lorena, University of São Paulo, P.O. Box 116, 12602-810 Lorena, SP, Brazil; ^2^Laboratory of Biocatalysis, Federal University of São João del Rei, P.O. Box 56, 35701-970 Sete Lagoas, MG, Brazil

## Abstract

Lipase from *Candida rugosa* (CRL) was immobilized by covalent attachment on hydrous niobium oxide. The matrix could effectively be attached to the enzyme with high retention of activity and prevent its leakage. Following immobilization, CRL exhibited improved storage stability and performed better at higher incubation temperatures. In addition, the enzyme retained most of its catalytic efficiency after successive operational cycles. The immobilized derivative was also fully characterized with respect to its morphological properties: particle size, surface specific area, and pore size distribution. Structural integrity and conformational changes, such as surface cavities in the support, set by the lipase procedure, were observed by Scanning Electron Microscopy. Additionally, a comparative study between free and immobilized lipases was provided in terms of pH, temperature, and thermal stability. CRL derivative was evaluated for the synthesis of biodiesel employing babassu oil and short chain alcohols. The process was feasible only for oil and butanol reaction system.

## 1. Introduction

Investigations of alternative renewable energy resources continue, with many studies focused on biodiesel fuels. One of the alternative renewable energy sources for diesel engines is vegetable oil. Studies have shown that pure vegetable oils are not a suitable direct replacement for diesel fuel, due to higher viscosity and molecular weight [1]. These properties cause incomplete combustion and engine deposits [2]. Therefore, several methods are used to reduce the viscosity of vegetable oil, such as transesterification reaction [3]. The transesterification reaction involves the reaction of alcohol with the oil, releasing glycerol as byproduct and monoalkyl esters (biodiesel), an excellent substitute for fossil fuels. In this reaction, alkali or acid are typically used as the catalysts resulting in high conversion levels in short reaction time [3, 4]. However, there are major drawbacks of such chemical process, as several problems during the steps of removal of catalyst from the product, recovery of glycerol, and the alkaline wastewater treatment, and interference of free fatty acid and water in the reaction and excessive consumption of energy [3, 4]. The use of heterogeneous catalysts, including immobilized lipases can overcome these problems which allow mild reaction conditions and no generation of chemical waste [3–7]. 

Lipases (E.C. 3.1.1.3) are ubiquitous enzymes with various biological activities, including triacylglycerols hydrolysis, esterification between fatty acid (FA) and alcohol, and other enzymatic reactions [8, 9]. Among the lipases from various sources, *Candida rugosa lipase *(CRL) received much attention due to its high activity and broad specificity [10, 11]. Published studies have demonstrated that CRL can be activated by interactions with a hydrophobic interface because of its conformational changes in its secondary structure (termed the “lid”) [11]. In the absence of a hydrophobic interface, the “lid” covers its active site and makes it inaccessible to substrates, whereas the conformational changes induced by interacting with the hydrophobic interface can yield an “open structure” of the lipase [12–14]. Thus, creating a hydrophobic microenvironment provides a preferable way to activate the lipase. Immobilization of lipase is expected to provide this hydrophobic microenvironment [13]. Moreover, enzyme immobilization provides a favorable approach to easy enzyme recovery for its recycled use and to extend its half-life. Thus, exploiting good immobilization carriers has been an attractive work for enzyme engineering. 

In this context, new inorganic matrixes as hydrous niobium oxides possess important properties of ionic exchange and show compatible affinity to be used as support for immobilizing chemical or biochemical catalysts.

Niobium is a rare, soft, malleable, ductile, gray-white metal with a body-centered cubic crystalline structure. Niobium is widely distributed in nature and it is about one and a half time as abundant as lead. It occurs in the minerals columbite and tantalite, together with tantalum. Niobium can form four oxides, from which the pentoxide, Nb_2_O_5_, is the basis of a series of salts called niobates and has wide applications including electronic and optical devices, special alloys, super conducting materials, and catalysts. Nb_2_O_5_ is also an excellent alternative for acid catalysis, acting as support, supported phase or associated with other metals (e.g., V, Pt, Mo, and W) increasing its selectivity toward many reactions. Hydrous niobium oxide is a polymeric material obtained from the hydrolysis of an intermediary product from alkaline fusion of Nb_2_O_5_ with an excess of K_2_CO_3_ [15].

In a previous work, hydrous niobium oxide was used as matrix for immobilizing the lipase from *Candida rugosa* by covalent binding in the presence of polyethylene glycol as stabilizing additive [16]. The immobilization conditions were established by factorial design and the resulted immobilized derivative showed considerable high hydrolytic and esterification activities that warranted the need for complementary studies concerning its morphological, biochemical, and kinetic properties. These studies are the objective of this paper.

In addition, the catalytic performance of the immobilized derivative was explored to carry out ester synthesis by direct esterification (butyl butyrate) and transesterification reaction using babassu oil (biodiesel). This oil is obtained from the kernels of the babassu palm, found in the northeast region of Brazil. It has broad application in cosmetic formulation [17–19]. The kernel contains 60–70% of a vegetable oil rich in saturated fatty acids lauric (C12) and myristic (C14), similar in composition to that of coconut and the use for to biodiesel production is still scarce related in the literature.

## 2. Materials and Methods

### 2.1. Materials

Commercial lipase from *Candida rugosa* (Type VII), bovine serum albumin (BSA), and *p*-nitrophenyl palmitate (*p*-NPP) were purchased from Sigma Chemical Co. (St Louis, MO, USA). This lipase is substantially free of other hydrolases and contains lactose as an extender. Nominal specific lipase activity was 1600 units·mg^−1^ of solid. Polyethylene glycol (PEG, MW 1,500, Merck, Germany) was used as stabilizing agent. *γ*-aminopropyltriethoxysilane (*γ*-APTS) was supplied by Across Organic (New Jersey, USA) and used without further purification. Gum arabic, Triton X-100, and heptane were supplied by Synth (SP, Brazil). Babassu oil was kindly supplied by COGNIS (Cognis from Brazil Ltda., Jacareí, SP, Brazil) having the following composition in fatty acids: (w/v): 3.5% caprylic, 4.5% capric, 44.7% lauric, 17.5% myristic, 9.7% palmitic, 3.1% stearic, 15.2% oleic, and 1.8% linoleic with average molecular weight 709.90 g/mol. Solvents were standard laboratory grade (Synth, São Paulo, SP, Brazil). Substrates for esterification reactions *n*-butanol (Merck, Germany) and butyric acid (Riedel-de Häen, Germany) were dehydrated, with 0.32 cm molecular sieves (aluminum sodium silicate, type 13 X-BHD Chemicals, Toronto, Canada), previously activated in an oven at 350°C for 6 h. Other reagents and solvents were of standard laboratory grade.

### 2.2. Preparation and Activation

Hydrous niobium oxide was prepared according to methodology previously established by Serafim [15] consisting of sintering niobium pentoxide with a fivefold excess by weight of potassium carbonate at 1000°C for 6 h, followed by addition of hot water and nitric acid 1.0 M. The material was dried at 50°C for 24 h. The support was submitted to a pretreatment with nitric acid (1%) at 75°C for 1 h to open the matrix pores and then silanized with *γ*-aminopropyltriethoxysilane (*γ*-APTS) and activated with glutaraldehyde at 2.5% and pH 8 according to the procedure established by Miranda et al. [16].

### 2.3. Immobilization Procedure 

Lipase was immobilized by covalent attachment on hydrous oxide niobium previously treated with *γ*-aminopropyltriethoxysilane (*γ*-APTS), followed by activation with glutaraldehyde [16]. For each gram of hydrous niobium oxide (dry weight) suitable amount of enzyme (0.25 g) was dissolved in distilled water and mixed with the support under low stirring during 3 h at room temperature. After this, 10 mL of hexane was added to the mixture enzyme support and the coupling took place overnight at 4°C. The derivative was filtered (Whatman filter paper 41) and thoroughly rinsed with hexane. Analyses of the hydrolytic activities carried out on initial and spent lipase solutions and immobilized preparations showed high level of lipase recovery on the support (*η* = 47.5%). 

### 2.4. Surface Area Determinations

The surface area measurements were performed by adsorption using nitrogen as adsorbate. The samples were previously degassed to below 50 mmHg at room temperature and the analyses were performed at 77 K using liquid nitrogen. The equilibrium interval was 5 sec. The surface area was calculated using the B.E.T. method. Pore volume and area distributions based on the BJH calculation were evaluated by the B.E.T apparatus software (NOVA 1200-Quantachrome).

### 2.5. Scanning Electron Microscopy (SEM)

Structural integrity and conformational changes, such as surface cavities in the support, set in by the lipase immobilizing procedure were observed by scanning electron microscopy (SEM) on Leica, LEO 440i.

### 2.6. Lipase Assay in Aqueous Solution

Hydrolytic activities of both free and immobilized lipase were measured with emulsified *p*-nitrophenyl palmitate (*p-*NPP) according to Kordel et al. [20]. One volume of a 16.5 mM solution of *p-*NPP in 2-propanol was mixed, just before, use with 9 volumes of 100 mM phosphate buffer pH 7.0 containing 0.4% (w/v) Triton X-100 and 0.1% (w/v) Arabic gum. Then, 2.7 mL of this mixture was pre-equilibrated at 37°C in a 1 mL cuvette of a UV-visible spectrophotometer (Varian UV-Carry, Varian Corporation). The reaction was started by addition of 0.3 mL of enzyme solution (at an appropriate dilution in 100 mM phosphate buffer pH 7.0) or 0.1 to 0.2 mg of immobilized lipase. The variation of the absorbance at 410 nm of the assay against a blank without enzyme was monitored for 5 min. Reaction rate was calculated from the slope of the curve absorbance versus time by using a molar extinction coefficient of 13.10^6^ cm^2^/mole for *p-*nitrophenol. This value was determined from the absorbance of standard solutions of *p-*NPP in the reaction mixture. One enzyme unit was the amount of enzyme liberating one *μ*mole of *p-*nitrophenol per minute in the above conditions.

### 2.7. Catalytic Properties of Free and Immobilized Lipase in Aqueous Medium

Free and immobilized hydrolytic activities were estimated with reaction mixtures containing 16.5 mM solution of *p*-NPP and 50 mM of sodium phosphate buffer at pH in the range from 6.0 to 8.5 at 37°C. The effect of temperature on lipase activity was determined at temperatures from 37 to 60°C for the free and immobilized lipase. For thermal stability tests, both free and immobilized lipase preparations were incubated in sodium phosphate buffer (pH 8.0 and 7.0, resp.) at different temperatures (40 to 60°C) for 1 h. Samples were removed and assayed for residual activity as previously described (Lipase assay in aqueous solution), taking an unheated control to be 100% active. The influence of substrate concentration on hydrolytic activities was also analyzed in *p*-NPP solutions at concentrations varying from 100 to 1000 *μ*M at 37°C. Values for *K*_*m*_ and *V*_max_ were calculated using the computational program Enzyme fitter version 1.05 published by Elsevier-Biosoft, 1987. 

### 2.8. Stability Tests

#### 2.8.1. Thermal Analysis 

Thermal gravimetric analysis was performed in a Shimadzu thermogravimetric instrument, TGA-50 model. Samples weighting 10 mg were examined at heating rates of 10°C·min^−1^ in a dry nitrogen flux from 30 to 600°C. 

#### 2.8.2. Stability Tests 

For thermal stability tests, both free and immobilized lipase preparations were incubated in 100 mM phosphate buffer (pH 8.0 and 7.0, resp.) at different temperatures (40 to 55°C) for 1 h. Samples were removed and assayed for residual activity as previously described (lipase assay in aqueous solution), taking an unheated control to be 100% active. Inactivation constants (*k*_*d*_) and half-life (*t*_1/2_) for both free and immobilized lipases were calculated according to (1) and (2), as shown below:

(1) $$\begin{array}{c} \ln\;A_{t}=\ln\;A_{0}-k_{d}\cdot t, \end{array}$$

(2) $$\begin{array}{c} t_{1/2}=\frac{\ln\;0.5}{-k_{d}}, \end{array}$$

where *A*_0_ is the initial activity, and *A*_*t*_ is the activity after heat treatment for *t* minutes.

The operational stability of the immobilized preparation was assayed in synthesis of butyl butyrate in successive batch runs. Reaction systems consisted of heptane (20 mL), *n*-butanol (100 mM), butyric acid (100 mM) and immobilized lipase in niobium oxide (0.5 g, d.wt). The mixture was incubated at 37°C for 24 h with continuous agitation at 150 rpm. At the end of each batch, the immobilized lipase was removed from the reaction medium and washed with hexane to remove any substrate or product retained in the matrix. Then, the immobilized lipase was introduced into a fresh medium. Reactions were monitored by measuring reactants and product concentrations by gas chromatography using a 6 ft 5% DEGS on Chromosorb WHP, 80/10 mesh column (Hewlett Packard, Palo Alto, CA, USA) and hexanol was used as an internal standard. Water concentrations in liquid and solid phases were measured by Karl Fischer (Mettler DL 18). Activities were estimated at the end of each cycle and expressed as *μ*mol·min^−1^·mg^−1^ of catalyst. The biocatalyst half-life (*t*_1/2_) was determined by applying the inverted linear decay model [21].

### 2.9. General Procedure for Biodiesel Synthesis

The reactions were performed in closed reactors with capacity of 25 mL, coupled with condensers, containing 12 g of substrate consisting of babassu oil and short chain alcohol (ethanol, propanol or butanol), without addition of solvents, at molar ratio oil to alcohol (1 : 10). The mixtures were incubated with *C. rugosa* immobilized in niobium oxide at proportions of 20% wt. in relation to the total weight of reactant involved in the reaction media. The experiments were carried out at temperature range from 40 to 50°C, depending on the reaction system, according to what was previously established in [6]. Reactions were performed for a maximum period of 96 h under constant magnetic agitation of 150 rpm. For the time course studies, an aliquot of reaction medium was taken at various time intervals and diluted in *n*-heptane for GC-analysis. 

### 2.10. GC Analysis

Samples prepared as described above were analyzed by injecting 1 *μ*L of heptane solution and internal standard into a GC (Varian Model 3800), equipped with a 6 ft 5% DEGS on Chromosorb WHP, 80/10 mesh column (Hewlett Packard, Palo Alto, CA, USA) following conditions previously established in [6]. All samples were measured in triplicate. Theoretical ester concentrations were calculated by taking into consideration the babassu oil fatty acid composition and its initial weight mass in the reaction medium [6, 7].

## 3. Results and Discussion 

### 3.1. Morphological Biocatalyst Characterization

**Figure 1:**
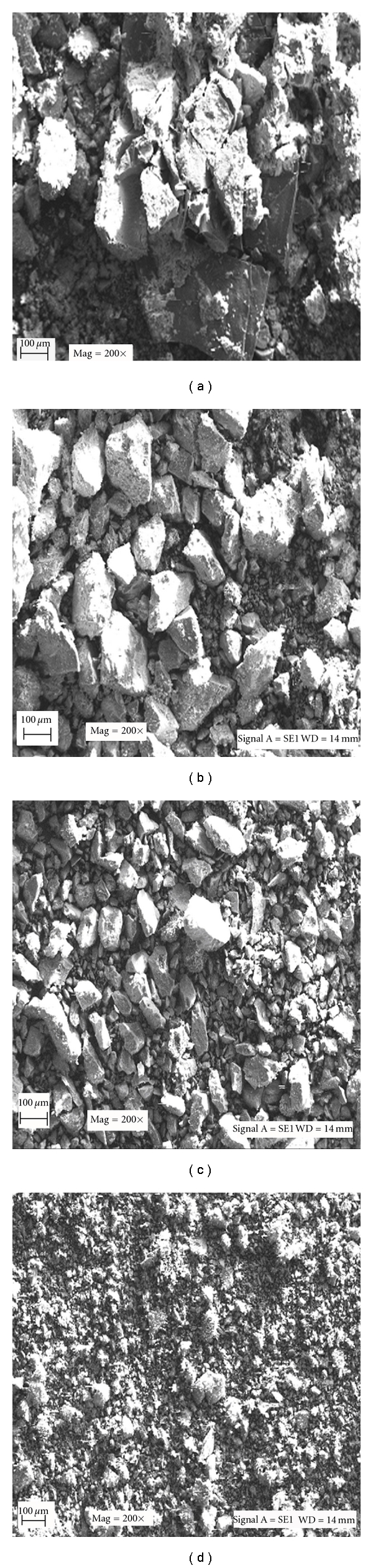
Scanning electron micrographs (SEM): (a) pure niobium oxide; (b) silanized niobium oxide; (c) silanized and activated niobium oxide; (d) immobilized CRL on niobium oxide.

The morphology, distribution, and size of the particles for pure, silanized, and activated niobium oxide and immobilized derivative were studied by micrographics obtained in scanning electronic microscope as presented in Figures 1(a), 1(b), 1(c), and 1(d). The method of B.E.T was also used as tool for evaluating the specific surface area, mean pore diameter, and pore volume average of these samples. 

Figure 1(a) shows that the pure niobium oxide is formed by particles having irregular size and surface showing different arrangements with a specific surface area of 42.15 m^2^/g and pore volume of 0.029 cm^3^/g. After the support silanization with *γ*-APTS (Figure 1(b)) the particles became more compacted, not showing any more empty spaces, a situation that also was confirmed by the specific surface area and pore volume values. With the incorporation of the silane group in the support surface and inside the pores, the specific surface area was reduced to 31.89 m^2^/g and pore volume to 0.024 cm^3^/g. Comparing the micrographs for niobium oxide silanized (Figure 1(b)) and activated with glutaraldehyde (Figure 1(c)), slight visual changes in the particle size and form were observed as further confirmed by the specific surface area and pore volume values, respectively, 33.57 m^2^/g and 0.027 cm^3^/g. The treatment with silane agent open the porous matrix and activation with glutaraldehyde reduced the surface area and pore volume due to the cross-linking reaction on the support surface. For the immobilized lipase on niobium oxide (Figure 1(d)), the micrograph shows that the support particles were fully covered by the enzyme and individual support particles cannot be any more visualized, which again was confirmed by the reduction on the specific surface area (10.08 m^2^/g) and the pore volume (0.011 cm^3^/g). 

In the immobilization procedure, another fundamental parameter is the support pore size, which should be sufficiently large to allow the enzyme accommodation. The niobium oxide silanized and activated with GA showed a pore volume of 0.027 cm^3^/g and the *Candida rugosa* lipase presents a molecular volume of 6.93 × 10^−20^ cm^3^ (5 × 4.2 × 3.3 nm^3^) [22], proving that the enzyme did not suffer any barrier to access the pore of the support. 

### 3.2. Biochemical and Kinetic Properties of the Immobilized Lipase on Niobium Oxide

**Figure 2:**
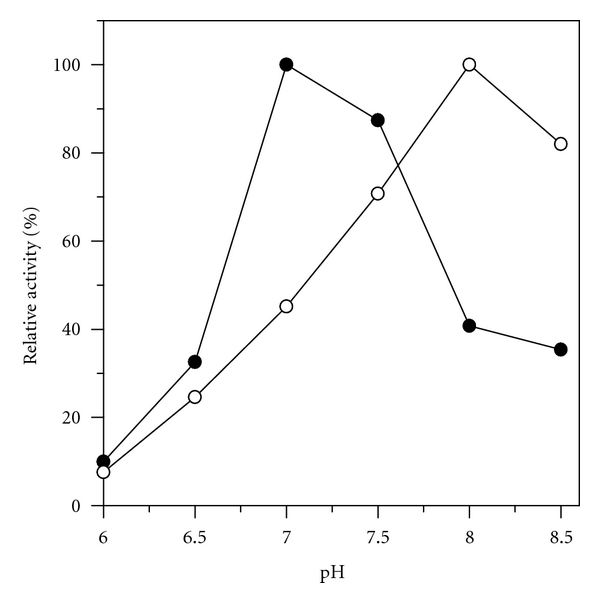
Effect of reaction pH on hydrolytic activities of lipase preparations. Enzymes were assayed with *p*-NPP as substrate at 37°C, (∘) free lipase, (●) niobium oxide-lipase. Starting activities (free lipase: 1553 units·mg^−1^; niobium oxide-lipase: 143 units·mg^−1^) were taken as 100%.

**Figure 3:**
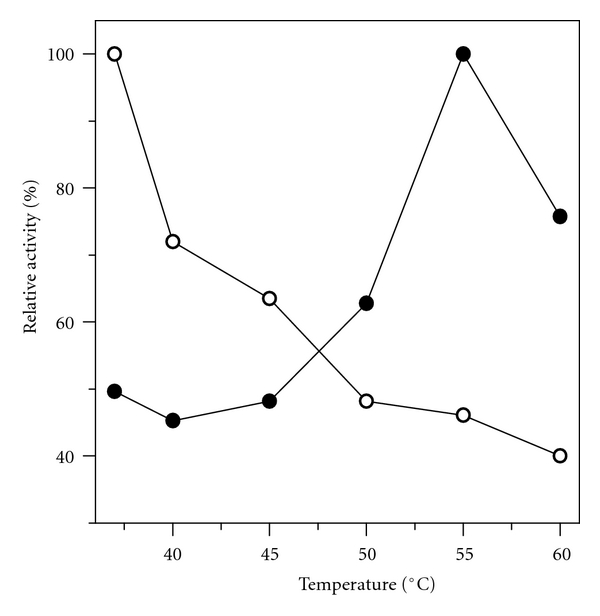
Effect of reaction temperature on hydrolytic activities of lipase preparations. Enzymes were assayed with *p*-NPP as substrate at pH 7.0, (∘) free lipase, (●) niobium oxide-lipase. Starting activities (free lipase: 1868 units·mg^−1^; niobium oxide-lipase: 271 units·mg^−1^) were taken as 100%.

The pH and temperature profiles of hydrolytic activities are shown in Figures 2 and 3, respectively. The pH optimum shifted 1.0 pH point from about 8.0 for the free enzyme, to about 7.0 for the immobilized lipase (Figure 2). However, this shift depended on the method of immobilization as well as the structure of the matrix, mainly the support charge surface. In relation to the support niobium oxide due to its positive charged, the optimal pH for immobilized lipase shifted to the acidic side. In the present work, however, care was taken to avoid the electrostatic potential within the immobilized lipase by carrying out the niobium oxide activation step in buffering pH solution (glutaraldehyde in phosphate buffer, 100 mM and pH 8.0), as described in Section 2. This was a necessary step since the charge groups on the support surfaces may induce acid or base catalyzed acyl migration that should be avoided during lipase-catalyzed process [23]. 

The optimum temperature for the immobilized enzyme (55°C) was 18°C higher than for the soluble enzyme (37°C), as shown in Figure 3. The increase in the optimum temperature of the immobilized lipase may be explained by the change of the conformational integrity of the enzyme structure upon covalent attachment to the support. The increase in the optimum temperature of the immobilized lipase is important for a possible industrial application because it allows to reduce microbial contamination and viscosity reduction of oil and greases, favoring high yield process, such as biodiesel production from vegetable oils. 

**Figure 4:**
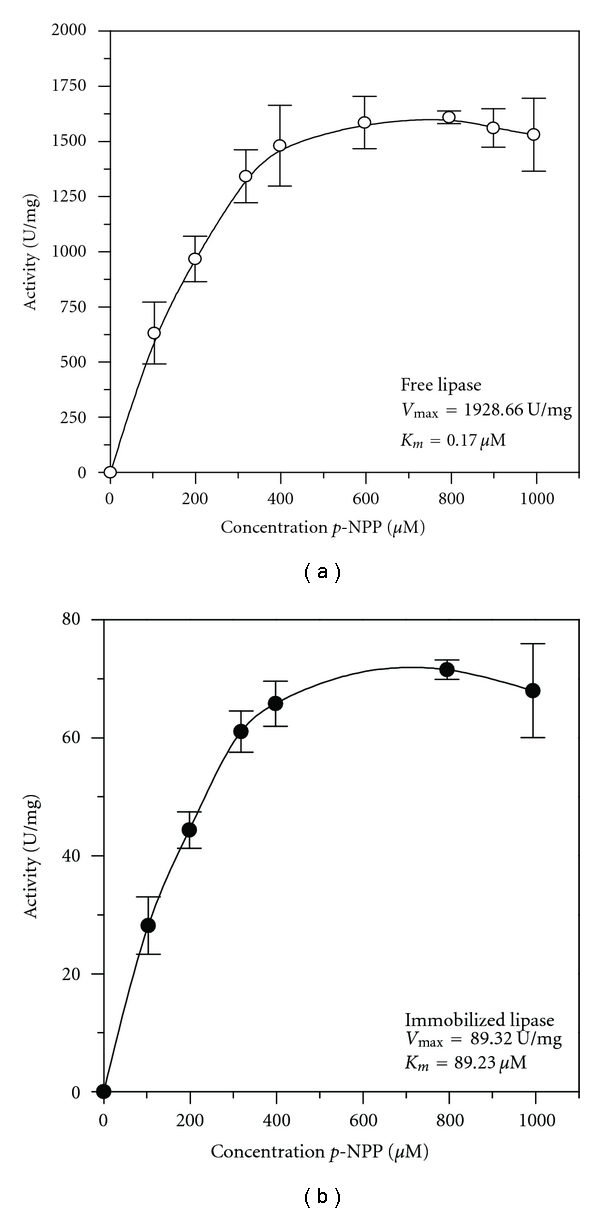
Hydrolytic activities for free lipase (a) and niobium oxide lipase (b) as a function of *p*-NPP concentration at pH 7.0 and 37°C.

The influence of substrate concentration on hydrolytic activities was also analyzed for free and immobilized lipase in *p*-NPP solutions varying from 100 to 1000 *μ*M (Figures 4(a) and 4(b)) to determine the Michaelis-Menten constant (*K*_*m*_) as the concentration of substrate at which half of the maximum reaction rate (*V*_max_) is reached. Plotting activity versus substrate concentration indicated that both lipases obey the Michaelis-Menten equation, indicating that in the range studied, no inhibition by reaction product was detected. The values obtained for *K*_*m*_ were 0.17 and 0.17 *μ*M for the free and immobilized lipase, respectively, indicating similar substrate affinity to the active site of the free and immobilized lipase. The maximum reaction rate (*V*_max_) for *p-*NPP hydrolysis with the free lipase was 1928.66 *μ*mol·mg^−1^·min^−1^ and 89.23 *μ*mol·mg^−1^·min^−1^ for the immobilized lipase. This decrease on the maximum reaction rate was either due to the conformational changes of the enzyme resulting in a lower possibility of forming a substrate-enzyme complex, or a less access of the substrate to the active sites of the immobilized enzyme caused by the increased diffusion limitation [23, 24]. In Table 1 the biochemical and kinetics parameters of the both free and immobilized lipase are summarized.

### 3.3. Stability Tests

#### 3.3.1. Thermal Stability

The thermal stability of enzymes is one of the important criteria for long-term and commercial application. The activity of immobilized enzyme is known to be more resistant against heat than native state. Thermal gravimetric analysis (TGA) provides an important tool for thermal stability studies of macromolecules [25, 26]. This technique enables to determine the temperature range at which a heated sample undergoes a major conformational change by means of monitoring the thermal weight loss profile. In the case of free lipase and immobilized lipase derivative such temperature range can be related to the protein unfolding and thus to the enzyme inactivation. Thermal degradation temperatures were determined for free lipase, hydrous niobium oxide, and the resulting immobilized lipase derivative. Thermal weight loss results are showed in Table 2. 

Free lipase was characterized by two weight loss peaks. In the first one, at temperature range from 30 to 180°C, distinguished by a low weight loss (4.3%) due to the dehydration of the interstitial water containing in the free lipase sample. From 180 to 600°C, continuous weight loss was observed indicating a complete decomposition of the organic structure of lipase.

The thermogravimetric analysis for the niobium oxide indicated a large weight loss (10.5%) at temperature lower than 200°C and a small one (4.9%) from 200 to 400°C. The former weight loss was due to the elimination of the free water and the latter to the water that it held in the support pores [26]. For the lipase immobilized on silanized and activated niobium oxide showed weight loss in two steps. The first was characterized by a weight loss (13.6%) at 30 to 200°C due to the elimination of hydration water and the second step was characterized by 5% weight loss at 200 to 400°C corresponding to water loss that is held in the pores. The results indicated that upon immobilization, the profile curves for lipase derivative shifted towards higher temperatures suggesting that a strong interaction between enzyme and the support occurred which enhanced the conformation stability of the native form. 

According to Table 3, free CRL at 55°C shows a half-life of 0.38 h, maintaining only 20% of its original activity after 1 h, whereas the half-life of the immobilized lipase at this temperature was 0.82 h. The conformational flexibility of the enzyme was reduced after immobilization [23, 24, 27, 28]. Immobilization of the lipase on niobium oxide led to an increase of enzyme rigidity, which protected it from unfolding. Thus the immobilized enzyme showed higher thermal stability than free enzyme, twice fold more stable than free lipase, similar stabilization factor (ratio between half-life of the immobilized and free lipase) observed at 50°C.

#### 3.3.2. Storage Stability

To complete the characterization of the immobilized lipase, the hydrolytic activity was measured as a function of long-term storage time at refrigerated temperature (4°C), and after 60 days the activity decreased by 80%. Results from literature, show that immobilized lipase from porcine pancreas (PPL) in chitosan stored in water and without any medium (dry), after four and ten days was verified total reduction of the hydrolytic activity of the immobilized derivates, respectively [29].

#### 3.3.3. Operational Stability

**Figure 5:**
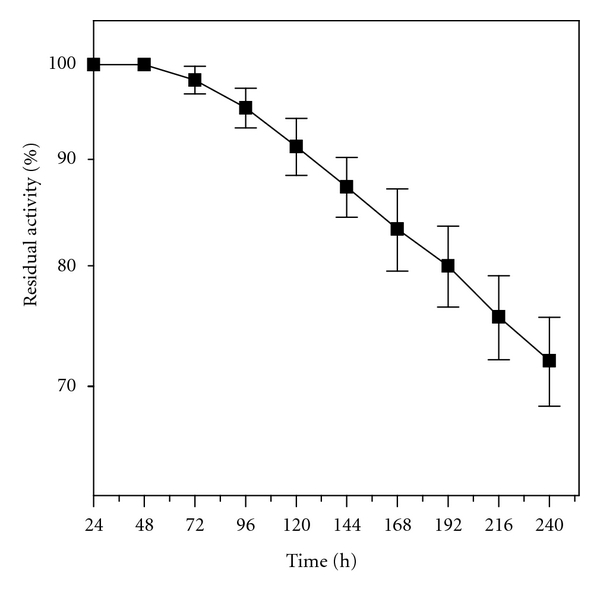
Batch operational stability tests for lipase immobilized in niobium oxide lipase in organic media. Initial activity (54.46 *μ*mol·g^−1^·min^−1^) was defined as 100%.

Considering that operational stability is a parameter of fundamental importance in developing processes with immobilized enzymes, complementary tests were performed to determine the biocatalyst half-life time for immobilized lipase in organic (ester synthesis) medium (Figure 5). For the esterification reaction of butanol with butyric acid (24 h-37°C), a slow decrease in the esterification activity was verified (25%) after ten recycles (240 h), which corresponds to a half-life (*t*_1/2_) of 406 h.

### 3.4. Application of the Prepared Derivative for Biodiesel Synthesis

**Figure 6:**
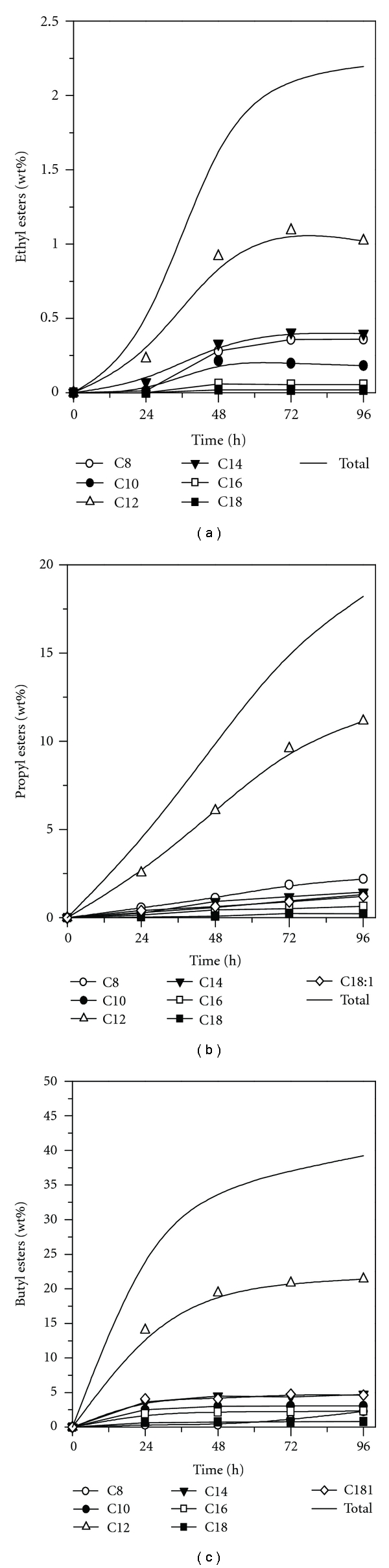
Profile for alkyl esters formation in the transesterification reaction of the babassu oil with ethanol (a), propanol (b), and butanol (c) catalyzed by CRL immobilized on niobium oxide as biocatalyst.

Transesterification reactions were performed using babassu oil and short-chain alcohol such as ethanol, propanol, and butanol catalyzed by *Candida rugosa* lipase on immobilized niobium oxide in solvent-free system. The long-chain fatty acid alkyl esters (FAAEs) produced (biodiesel) can be used as substitute to the conventional diesel fuel without any modification in engine design [3, 4].  Figure 6 shows the progress of the enzymatic reaction for each system in terms of monoalkyl esters formation. 

According to the results, *Candida rugosa* lipase was strongly inactivated when ethanol was used as acyl acceptor (transesterification yield = 4.36%), a result which is in agreement with those related in the literature [30, 31]. Conversion of babassu oil into fatty acid alkyl esters (FAAEs) was also affected by propanol reaching a yield lower than 40%. The lower performance into fatty acid alkyl esters formation when ethanol and propanol were used as acyl acceptors can be attributed to the high polarity of these alcohols, which leads to an unfavored partition of water between enzyme and support, thus stripping essential water from enzyme molecules and reducing the CRL activity. Enzymatic activity and stability are highly dependent on the degree of hydration of the enzyme and a minimum amount of water is necessary for the protein to maintain its optimal conformation [30, 31].

Therefore, in the conversion of babassu oil into alkyl esters butanol gave the highest conversion (79.35%), showing a better activity of the *Candida rugosa* lipase with increasing alcohol chain lengths due to the lowest polarity of the butanol in relation to the ethanol and propanol. Similar results were reported by Cambon et al. [31] in the transesterification of sunflower oil catalyzed by vegetal lipase.

### 3.5. Economical Evaluation for the Manufacture of the Immobilized Lipase on Niobium Oxide

The use of enzymes as industrial catalysts for replacing the conventional chemical processes is limited mainly by the high biocatalyst and its low operational stability. The immobilization in appropriate supports can overcome such limitation since the initial investment in raw material (enzyme, support, and reagents) is expected to be rewarded by the high activity and stability of the resulted immobilized derivative [13, 16]. 

To estimate the overall cost for preparing the immobilized lipase on niobium oxide, it was taken into consideration a batch starting from 100 g of pure niobium oxide and the reagent costs, including the free lipase as stated in catalog prices directed available from the manufactures. The involved cost for the immobilizing procedure to obtain immobilized derivative on controlled pore silica (US$ 67.0/g) was used for comparative purpose. The cost of *Candida rugosa* lipase immobilized on niobium oxide was estimated to be 1.06 US$/g immobilized system, corresponding to value 64 times lower than one obtained by the immobilization derivative of that same lipase on controlled pore silica (US$ 68.0/g immobilized system). Considering that the lipase immobilized on niobium oxide showed similar values for thermal and operational stabilities to that one obtained for the lipase immobilized on commercial inorganic matrix, as controlled pore silica [32], it can be inferred that the proposed matrix according to the description given in this work can be replaced with technical and economical advantages of commercial inorganic matrixes, as controlled pore silica. For a better evaluation of the properties obtained by the lipase immobilized on niobium oxide, comparative data described in the literature for experimental preparations of *Candida rugosa* immobilized on inorganic matrixes, as zirconium phosphate (ZrP), controlled pore silica (CPS) and silica-gel obtained by the sol-gel technique using MTMS as precursor [24, 32, 33] is given in Table 4. 

In terms of activity recovery and operational stability, the matrix used in the present work shows comparable performance with other inorganic matrixes, indicating that niobium oxide can be used as an alternative to commercial matrices, such as controlled pore silica.

## 4. Conclusion

The immobilized derivative obtained under the conditions previously established was fully characterized with respect to its morphological properties: particle size, surface specific area, and pore size distribution (B.E.T. method) and yield of grafting (TGA). Structural integrity and conformational changes, such as surface cavities in the support, set by the lipase procedure were observed by scanning electron microscopy (SEM). The catalytic performance of the immobilized derivative was also determined in aqueous (hydrolysis of ester) and in nonaqueous media (synthesis of ester and modification of vegetable oil). In aqueous media, a comparative study between free and immobilized lipase was made in terms of pH, temperature, thermal stability, and kinetics parameters using *p*-phenyl palmitate as substrate. Upon immobilization the lipase shifted optima conditions for lower pH (7.00) and higher temperature (55°C) values when compared with the lipase in its free form (pH 8.0 and temperature of 37°C). Thermal stability tests revealed that the immobilized derivative showed higher thermal stability than the free lipase. The influence of the substrate concentration on the reaction rate showed good adjustment of Michaelis Menten model, with an estimation of the same *K*_*m*_ values for both free and immobilized enzymes (0.17 *μ*M) and *V*_max_ values of 1928.66 U/mg for free and 89.23 U/mg for immobilized lipase. 

In nonaqueous media, the catalytic potential of the immobilized derivative was verified in the esterification reaction of butanol with butyric acid (operational stability) and transesterification reaction of babassu oil with short chain alcohols. In the operational tests, the immobilized derivative was used successively under batch reactions (24 h/37°C) maintaining up to 70% of the initial activity up to 240 h, revealing a biocatalyst half-life of 406 h. CRL was inhibited when ethanol and propanol were used as acyl donors in the transesterification of babassu oil into biodiesel. Therefore, the immobilized lipase was shown to be only effective for the transesterification with butanol, reaching satisfactory concentrations of butyl esters.

## Figures and Tables

**Table 1:** Biochemical and kinetics properties of the free and immobilized lipase.

Parameter	Free lipase	Immobilized lipase
Optimum pH	8.0	7.0
Optimum temperature (°C)	37	55
*K*_*m*_ (*μ*M)	0.17	0.17
*V*_max _ (U/mg)	1928.66	89.23

**Table 2:** Thermogravimetric analysis for support, free lipase, and immobilized derivative.

Material	Temperature range (°C )	Thermal degradation temperature* (°C)	Weight loss ( % )
Pure support	30–200	53	10.5
	200–400		4.9
Free lipase	30–180	52	4.3
Immobilized lipase	30–200	54	13.6
	200–400		5.0

*Temperature at which a heated sample undergoes a major weight loss.

**Table 3:** Half-life (*t*_1/2_) and rate of denaturation (*k*_*d*_) for the free and immobilized lipases.

Temperature (°C)	Rate of denaturation (*k*_*d*_) (h^−1^)		Half-life (*t*_1/2_) = −ln (1/2)/*k*_*d*_ (h)	
	Free lipase	Immobilized lipase	Free lipase	Immobilized lipase
40	0.23	0.22	3.03	3.15
50	1.15	0.52	0.60	1.36
55	1.81	0.84	0.38	0.82

**Table 4:** Comparison of the catalytic properties of the *Candida rugosa* lipase immobilized in different inorganic supports.

Property	Support			
	CPS^a^	Silica^b^ (sol-gel)	ZrP^c^	Nb_2_O_5_
Activity recovered (%)	48	1.34	60	47.5*
Hydrolytic activity (U/mg)	119	4.98	163	124*
Esterification Activity (*μ*M/g·min)	154	136	103	120*
Operational stability in organic medium (*t_1/2_* at 37°C, h)	417	132	412	406
References	[32]	[33]	[24]	This work

^a^ CPS: Controlled pore silica.

^b^encapsulated lipase in hydrophobic matrix obtained by sol-gel process using as precursor MTMS (methyltrimethoxysilane).

^c^ZrP: zirconium phosphate.

*results previously reported [16].
